# Effect of Dietary and Lifestyle Interventions on the Amelioration of NAFLD in Patients with Metabolic Syndrome: The FLIPAN Study

**DOI:** 10.3390/nu14112223

**Published:** 2022-05-26

**Authors:** Sofía Montemayor, Cristina Bouzas, Catalina M. Mascaró, Miguel Casares, Isabel Llompart, Itziar Abete, Escarlata Angullo-Martinez, María Ángeles Zulet, J. Alfredo Martínez, Josep A. Tur

**Affiliations:** 1 Research Group on Community Nutrition and Oxidative Stress, University of the Balearic Islands—IUNICS, 07122 Palma de Mallorca, Spain; sofiamf16@gmail.com (S.M.); cristina.bouzas@uib.es (C.B.); c.mascaro@uib.es (C.M.M.); isabel.llompart@ssib.es (I.L.); eangullo@ibsalut.caib.es (E.A.-M.); 2 Health Institute of the Balearic Islands (IDISBA), 07120 Palma de Mallorca, Spain; 3 CIBEROBN (Physiopathology of Obesity and Nutrition CB12/03/30038), Instituto de Salud Carlos III (ISCIII), 28029 Madrid, Spain; iabetego@unav.es (I.A.); mazulet@unav.es (M.Á.Z.); 4 Radiodiagnosis Service, Red Asistencial Juaneda, 07011 Palma de Mallorca, Spain; casaresmiguel@gmail.com; 5 Clinical Analysis Service, University Hospital Son Espases, 07120 Palma de Mallorca, Spain; 6 Department of Nutrition, Food Sciences, and Physiology, Center for Nutrition Research, University of Navarra, 31008 Pamplona, Spain; jalfredo.martinez@imdea.org; 7 Escola Graduada Primary Health Care Center, 07001 Palma de Mallorca, Spain; 8 Cardiometabolics Precision Nutrition Program, IMDEA Food, CEI UAM-CSIC, 28049 Madrid, Spain

**Keywords:** NAFLD, metabolic syndrome, physical activity, intrahepatic fat contents, Mediterranean diet, FLIPAN

## Abstract

Background: Adults with fatty liver present unusual glycaemia and lipid metabolism; as a result, non-alcoholic fatty liver disease (NAFLD) is now considered as part of the metabolic syndrome (MetS). Objective: To assess the 6- and 12-month effects of customized hypocaloric dietary and enhanced physical activity intervention on intrahepatic fat contents and progression of NAFLD, in patients with MetS. Design: Cross-sectional study in 155 participants (40–60 years old) from Balearic Islands and Navarra (Spain) with a diagnosis of NAFLD and MetS, and BMI (body mass index) between 27 and 40 kg/m^2^; patients were randomized in a 1:1:1 ratio to either Conventional Diet, Mediterranean diet (MD)–high meal frequency, and MD–physical activity groups. Methods: Dietary intake was assessed using a validated food frequency questionnaire. Adherence to Mediterranean diet, anthropometrics, physical activity, and biochemical parameters (fasting glucose, glycated hemoglobin, bilirubin, aspartate aminotransferase, alanine aminotransferase—ALT–, gamma-glutamyl transferase, uric acid, urea, creatinine, albumin, total cholesterol, high-density lipoprotein cholesterol—HDL-cholesterol–, and triglycerides) were also assessed. Results: Subjects with NAFLD and MetS had reduced intrahepatic fat contents, and liver stiffness, despite the intervention the participants went through. All participants ameliorated BMI, insulin, Hb1Ac, diastolic blood pressure, HDL-cholesterol, and ALT, and improved consumption of total energy, fish, and legumes. Participants in the MD–HMF group improved waist circumference. Conclusions: Customized hypocaloric dietary and enhanced physical activity interventions may be useful to ameliorate NAFLD.

## 1. Introduction 

NAFLD is characterized by hepatocyte triacylglycerol accumulation (steatosis), which can progress to inflammation, fibrosis, and cirrhosis (steatohepatitis) [1]. Increased lipogenesis, together with hyperlipidemia and raised fat deposition, contribute to non-alcoholic fatty liver disease [2].

Adults as well as children with fatty liver display abnormal glucose and lipid metabolism; as a result, NAFLD is now regarded as a key part of the MetS [3]. The ‘multiple hit’ hypothesis, including hits such as insulin resistance, lipotoxicity, nutritional factors, gut microbiota, genetic and epigenetic factors, is currently the most widely accepted theory for NAFLD pathogenesis [3]. Lifestyle modifications, such as exercise and a healthy diet, resulting in sustained weight loss, are the only proven and effective strategies available currently to curtail the NAFLD burden [2]. While weight loss is the single most effective intervention, it can be extremely challenging for patients to achieve the sustained weight loss goal [4]. Currently, there is no consensus concerning the pharmacological treatment of NAFLD. The cornerstone of NAFLD management is, however, lifestyle interventions focused on exercise and a well-balanced diet for both quality and quantity [5]. Despite some between-country variations, the Mediterranean diet (MedDiet) in Spain reflects a food consumption pattern rich in fruits and vegetables, legumes, whole grains, olive oil, fish and nuts, with greater consumption of white or lean meats than of red or processed meats, moderate consumption of dairy products, and intake of small amounts of wine, mainly red wine and with meals; many studies suggest that the protective effects of the MedDiet may be due mostly to the anti-inflammatory and antioxidant properties of its components [6]. NAFLD is associated with visceral obesity, insulin resistance, dyslipidemia, and chronic inflammation, all of which are features of MetS, and the MedDiet may improve NAFLD by modulating the presence of these conditions [7]. Evidence from limited epidemiological data indicates that greater adherence to the MedDiet is associated with lower probability of having non-alcoholic steatohepatitis (NASH) [8]. The present study aims to evaluate 6- and 12-month effects of customized nutritional interventions on the amelioration of intrahepatic fat deposits and progression of NAFLD among obese patients with metabolic syndrome. 

## 2. Methods 

### 2.1. Design 

The current study was a multicenter (Mallorca and Navarra, Spain) prospective randomized trial, with personalized nutritional intervention based on a Mediterranean diet, to evaluate whether and to which extent customized dietary and physical activity intervention ameliorate NAFLD among obese patients with Metabolic Syndrome. Inclusion criteria were aged 40–60 years, Body Mass Index (BMI) between 27 and 40 kg/m^2^, NAFLD diagnosed by magnetic resonance imaging (MRI; Signa Explorer 1.5T, General Electric Healthcare, Chicago, IL, USA), and with MetS traits as described by the International Diabetes Federation (IDF) consensus [9]. Exclusion criteria were previous cardiovascular disease, congestive heart failure, liver diseases (other than NAFLD), cancer or a history of malignancy in the previous 5 years, previous bariatric surgery, acute febrile illnesses, urinary tract infections, post-renal hematuria, hemochromatosis, protein overload, non-medicated depression or anxiety, alcohol and drug abuse, pregnancy, primary endocrinological diseases (other than hypothyroidism and type 2 diabetes mellitus), concomitant therapy with steroids, intense physical exercise, or being unable to provide informed consent. 

### 2.2. Ethics

The study was registered at ClinicalTrials.gov with number NCT04442620 (https://clinicaltrials.gov/ct2/show/NCT04442620; accessed on 25 February 2022). The study protocol was reviewed and approved by the Ethics Committee of the Balearic Islands (ref. IB 2251/14 PI; approved on 26 February 2020) and the Ethics Committee of the University of Navarra (ref. 054/2015mod2; approved on 22 February 2018), and it followed the Declaration of Helsinki ethical standards. 

### 2.3. Subjects

**Figure 1:**
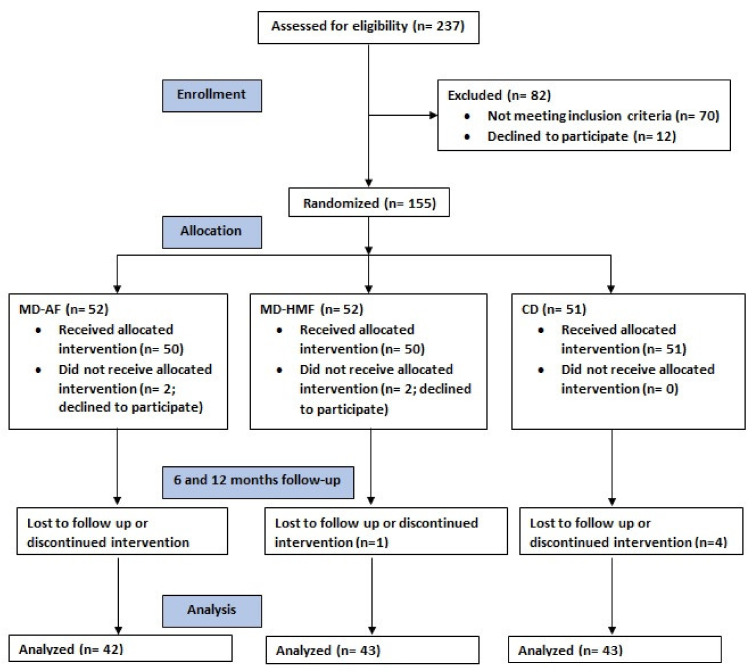
Flowchart of the study.

From June 2018 to January 2020, 237 patients were screened for eligibility. Of those, 82 did not meet inclusion criteria or refused to participate. Patients satisfying the selection criteria will be grouped according to the followings: stage of NAFLD 1, 2 or 3, whether have T2DM (yes/no), and gender. Finally, 155 patients were randomized using the MinimPy desktop minimization program in a 1:1:1 ratio to one of the three intervention groups for 12 months: Conventional diet (CD), AASLD recommendations; MedDiet–high meal frequency (MD–HMF); and MedDiet–physical activity (MD–PA). Of the 155 patients included, 5 patients withdrew their consent before receiving intervention, and 22 withdrew their consent or were lost to follow-up before completing 6 and 12 months of the trial. Finally, data from a total of 128 participants were analyzed. Patients were distributed as follows: 43 in the CD group; 43 in the MD–HMF group; and 42 in the MD–PA group. A flowchart of recruitment and randomization is shown in Figure 1.

The intervention groups sought to ameliorate NAFLD among obese patients with Metabolic Syndrome. Three interventions were chosen based on the conditions of the studied population. For the management of NAFLD, several international guidelines endorse weight loss either through a combination of dietary modifications and physical activity or through one of these [10,11]. Nevertheless, quality of diet composition could also play a crucial role. The MedDiet, naturally rich in antioxidants and anti-inflammatory foods, together with personalized physical activity could have a positive health effect on liver steatosis [8]. Hence, in the current study, a personalized nutritional intervention based on an energy-restricted customized Mediterranean diet, which introduces plenty of antioxidant and anti-inflammatory bioactive components, coupled with physical activity promotion, was considered as the control group and was compared with two more dietary strategies, which included a Mediterranean Diet intervention with seven meals a day, and the conventional dietary approach proposed by the American Association for the Study of Liver Diseases (AASLD).

The Conventional Diet (CD) group followed the American Association for the Study of Liver Disease (AASLD) recommendations [12] that recommends an energy restriction enough to lose 3–5% of body weight to improve steatosis, and 7–10% to improve most of the histopathological features of NAFLD/NASH, including fibrosis, following the general guidelines of the U.S. Department of Health and Human Services and the U.S. Department of Agriculture (20–35% fat, 10–35% protein, 45–65% carbohydrate) [13], and maintain an adequate fiber (25 g/day) and cholesterol (<250 mg/day) intake. Moreover, this group was instructed to accumulate a minimum of 10,000 steps a day (recorded by a personal pedometer) [14].

The Mediterranean Diet–high meal frequency (MD–HMF) group adhered to a MedDiet based on a distribution of macronutrients of 30–35% fat (mainly mono- and poly-unsaturated fatty acids from extra virgin olive oil, nuts, and omega-3 containing foods), 25% protein (mainly from vegetable sources), and 40–45% carbohydrates (50–70% of the total carbohydrate intake should be low glycemic index and rich in fiber). Patients allocated this treatment were advised to consume 7 meals a day, gradually reducing the caloric content at each main meal, with the highest calorie meals to be consumed early during the day (breakfast, lunch, dinner and two snacks in the morning and two snacks in the afternoon). Moreover, this diet was previously observed to reduce fat mass and overall weight and improve general oxidative stress in patients with metabolic syndrome [15], providing high Total Antioxidant Capacity (TAC), and focused on the chronological distribution of meals, as factors such as meal frequency and distribution could aid in reducing the feeling of hunger, thus improving compliance to an energy-restricted dietary regime [16]. Additionally, with the previous group, subjects were instructed to accumulate a minimum of 10,000 steps a day also recorded by a personal pedometer [17].

The Mediterranean Diet–physical activity (MD–PA) group also followed an energy-restricted MedDiet. However, meal frequency was of 4–5 meals a day including snacks. This group consumed 35–40% of total calories from fat (8–10% of saturated fatty acids, >20% of monounsaturated fatty acids, >10% of polyunsaturated fatty acids and <300 mg/day of cholesterol), approximately 20% of total calories from proteins and 40–45% or more of total calories from carbohydrates (low glycemic index). Sodium chloride should not exceed 6 g a day (2.4 g of sodium), and dietary fiber should be no less than 30–35 g/day [17]. Participants in this group, on the other hand, were instructed to undergo a 35 min interval training session three times a week, with a combination of two instructor-led on-site training sessions and one remote prescribed training session a week for the whole duration of the trial. Physical activity sessions of 35 min consisted of 5 min warm-up, 20 min interval training, and 10 min breathing and stretching. It has been pointed out that an association between physical activity and risk of nonalcoholic fatty liver disease, and people with NAFLD showed low levels of PA [18]. Three to five sessions per week of moderate or vigorous physical activity, an equivalent of 150–200 min per week, have been shown to decrease the development of NAFLD [19]. 

### 2.4. Dietary Intake Assessment

Each intervention aimed at reducing caloric intake by 25–30% of baseline intake and increasing energy expenditure by 400 kcal/70 kg of body weight (5.7 kcal per kg of body weight). Trained dietitians provided patients a daily calorie prescription, food regimens based on exchange systems, and a seven-day meal plan for each season by trained dietitians. Patients were instructed to weigh themselves weekly, and patient–dietitian contact (in person, by phone, or via e-mail) was offered once every two weeks for the first six months to aid adherence.

Information about intakes was collected at baseline and 6 months using a validated 148-item Food Frequency Questionnaire (FFQ) [20]. The 148 items consist of usual portion sizes of foods and drinks with response categories to indicate frequency of consumption over a period of 6 and 12 months; participants were asked how often, on average, they consumed the amount of item reported on the FFQ during the past year and respond using nine options ranging from never or less than once per month to six or more times per day. Additional foods not included in the questionnaire and the frequency of consumption were manually entered. Energy and nutrients intakes were calculated by multiplying the nutrient composition of the portion size of each item by the frequency of consumption using a computer program based on available food composition tables [21]. Dietary information derived from the 148-item FFQ included total energy expressed as kcal per day (kcal/d), macro- and micro-nutrient intakes, and intakes according to food groups.

Adherence to the MedDiet was assessed by means of a previously used, validated 17-item MedDiet adherence questionnaire [22]. A score was given for each met objective: 1 (compliance) or 0 (non-compliance). The total score ranged between 0 and 17, with a score of 0 indicating no compliance, and a score of 17 indicating maximum adherence.

### 2.5. Anthropometrics

At baseline and at 6 and 12 months follow-up, weight, body fat, BMI, waist circumference (WC), blood pressure (BP), and information on energy expenditure were taken by trained dietitians. Height was measured at baseline using a mobile stadiometer (Seca 213, SECA Deutschland, Hamburg, Germany), with the participant’s head maintained in the Frankfort plane, and to the nearest millimeter. Weight and body fat were measured using a Segmental Body Composition Analyzer for impedance testing (Tanita MC780P-MA, Tanita, Tokyo, Japan), with participants wearing light clothes and no shoes (0.6 kg of weight was subtracted for their clothing). Using a measuring tape, the waist circumference was taken halfway between the last rib and the iliac crest, with participants standing upright. BMI was calculated as weight in kg/height in m^2^, using the standard formula (weight in kilograms divided by the square of height in meters). BP was measured in triplicate (2 min apart) with a validated semi-automatic oscillometer (Omron HEM-705CP, Hoofddorp, The Netherlands), in the non-dominant arm after a 5 min rest in a seated position. The average of the three measurements was recorded and used for statistical analysis. Information on mean weekly time (in minutes) of physical activity was collected using the Minnesota Leisure Time Physical Activity Questionnaire (Spanish version); energy expenditure was expressed as metabolic equivalents of task (MET) min/week [23,24].

### 2.6. Blood Collection and Biochemical Analysis

At each visit, venous blood and single spot urine samples were collected in the morning after a 12 h overnight fast. Blood was collected through a venous catheter from the antecubital vein in suitable vacutainers. Measures included routine biochemical parameters, such as fasting glucose, glycated hemoglobin (HbA1c), aspartate aminotransferase (AST), alanine aminotransferase (ALT), gamma-glutamyl transferase (GGT), total cholesterol, high-density lipoprotein cholesterol (HDL-C), and triglyceride (TG); these were measured in serum on the Abbott ARCHITECT c16000 employing commercial kits (Abbott Diagnostics, IL, USA). Low-density lipoprotein cholesterol (LDL-C) was calculated according to the Friedewald formula [25]. 

### 2.7. Diagnosis of NAFLD

Liver fat was verified by abdominal MRI (Signa Explorer 1.5T, General Electric Healthcare, Chicago, IL, USA) and quantified as mean percentage (%). A mean intrahepatic fat ≥6.4% was considered clinically relevant [26]. Elastography/Fibroscan on the other hand provided an estimation of liver stiffness that in turn is affected by fat infiltration [27].

### 2.8. Statistics

Sample size was estimated for weight loss as the primary outcome of the study, assuming a two-group *t*-test (two-sided) of the difference between the control group and the two intervention groups (group ratio = 2). Based on previous evidence [15,28], a weight reduction difference of 2.5 kg with a standard deviation (SD) of 4.5 was expected between the control group and the intervention groups. A total sample size of 150 patients was needed to give the trial a 95% power to detect a statistically significant difference in weight loss between the control and the intervention groups (α = 0.05), as well as to account for a 20% dropout rate. The analysis was conducted by modified intention to treat (mITT) with randomized participants analyzed according to the treatment group. Statistical analysis was performed using the SPSS statistical software package, version 27.0 (SPSS Inc., Chicago, IL, USA). Descriptive statistics were used for characteristics of the subjects. Continuous variables were expressed as means ± SD. Categorical data were expressed as count and percentage. To compare percentages, a Chi-square test was used. To compare values of quantitative variables with normal distribution, ANOVA was used. Changes (delta) in anthropometric, biochemical, and dietary parameters were evaluated by a paired t-test. Between-group changes were assessed by one-way ANCOVA, while statistically controlling for baseline measures. Post hoc analyses were performed by applying the Bonferroni method. Results were considered statistically significant if *p*-value < 0.05. 

## 3. Results 

Table 1 shows the characteristics of participants at baseline. No differences between all the groups (CD, MD–HMF, and MD–PA) were registered for any of the considered sociodemographic and clinical parameters.

**Figure 2:**
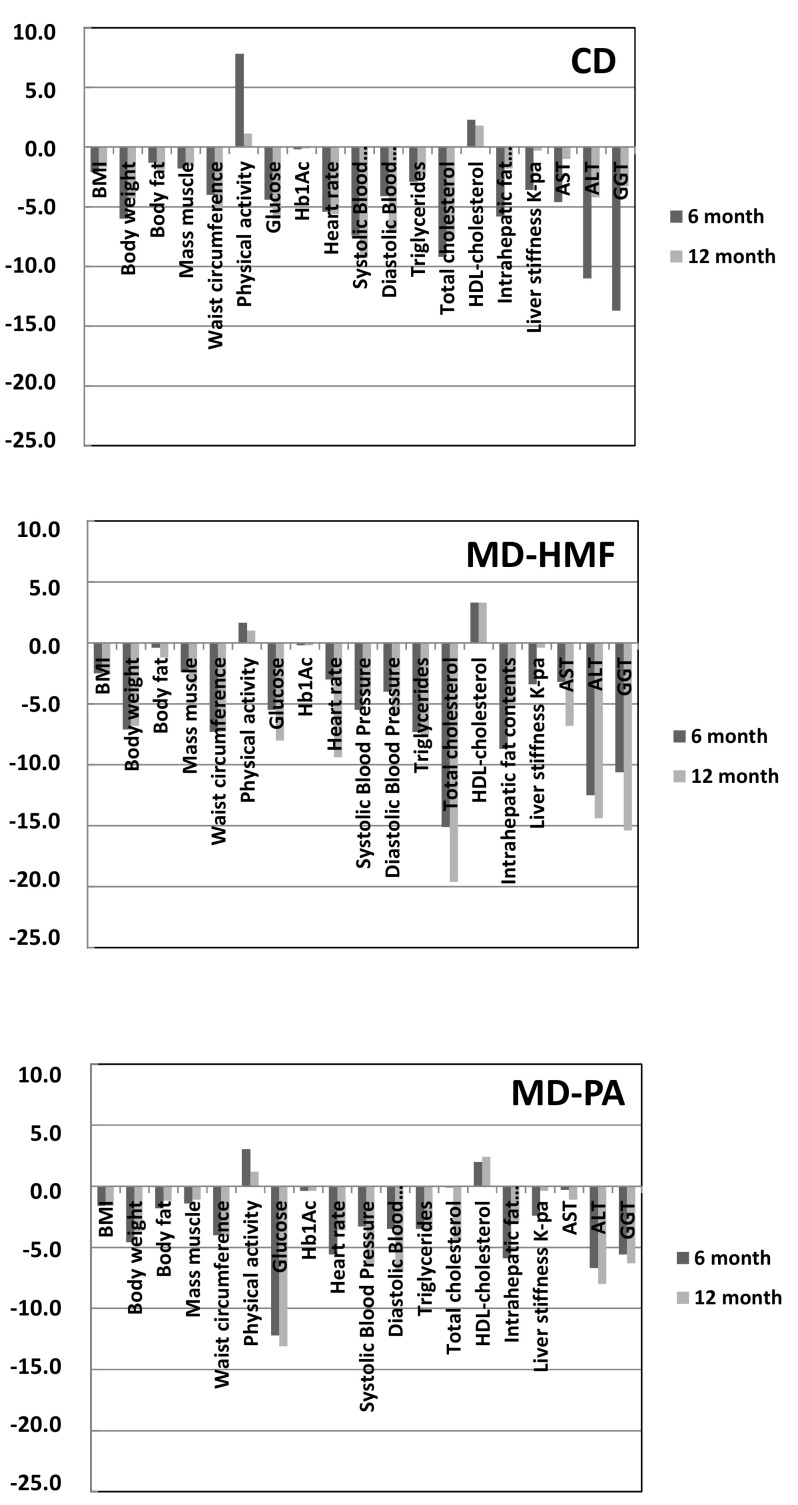
Changes in anthropometric, metabolic, and liver parameters at 6 and 12 months follow-up versus baseline. CD: Conventional Diet; MD–HMF: Mediterranean diet–high meal frequency; MD–PA: Mediterranean diet–physical activity (control group). Physical activity was represented as MET × 10^−2^. Triglycerides were represented as mg/dL.

Table 2 shows the anthropometric, metabolic, and liver parameters in the 6-month trial versus baseline. Concerning the anthropometric and metabolic parameters, BMI, body weight, insulin, Hb1Ac, diastolic blood pressure and HDL-cholesterol ameliorate in time with no difference between the three groups. In contrast with WC, the groups MD–PA and MD–HMF decreased their values (−4.0 ± 6.3 *p* < 0.001; −7.3 ± 5.8 *p* < 0.001), respectively, having differences between the groups MD–PA and MD–HMF (*p* = 0.046). Other parameters experienced changes through time in some treatment groups; however, no time*group interaction was found for parameters other than waist circumference. Changes in anthropometric, metabolic, and liver parameters at 6 and 12 months follow-up versus baseline can be visualized clearer in Figure 2.

**Figure 3:**
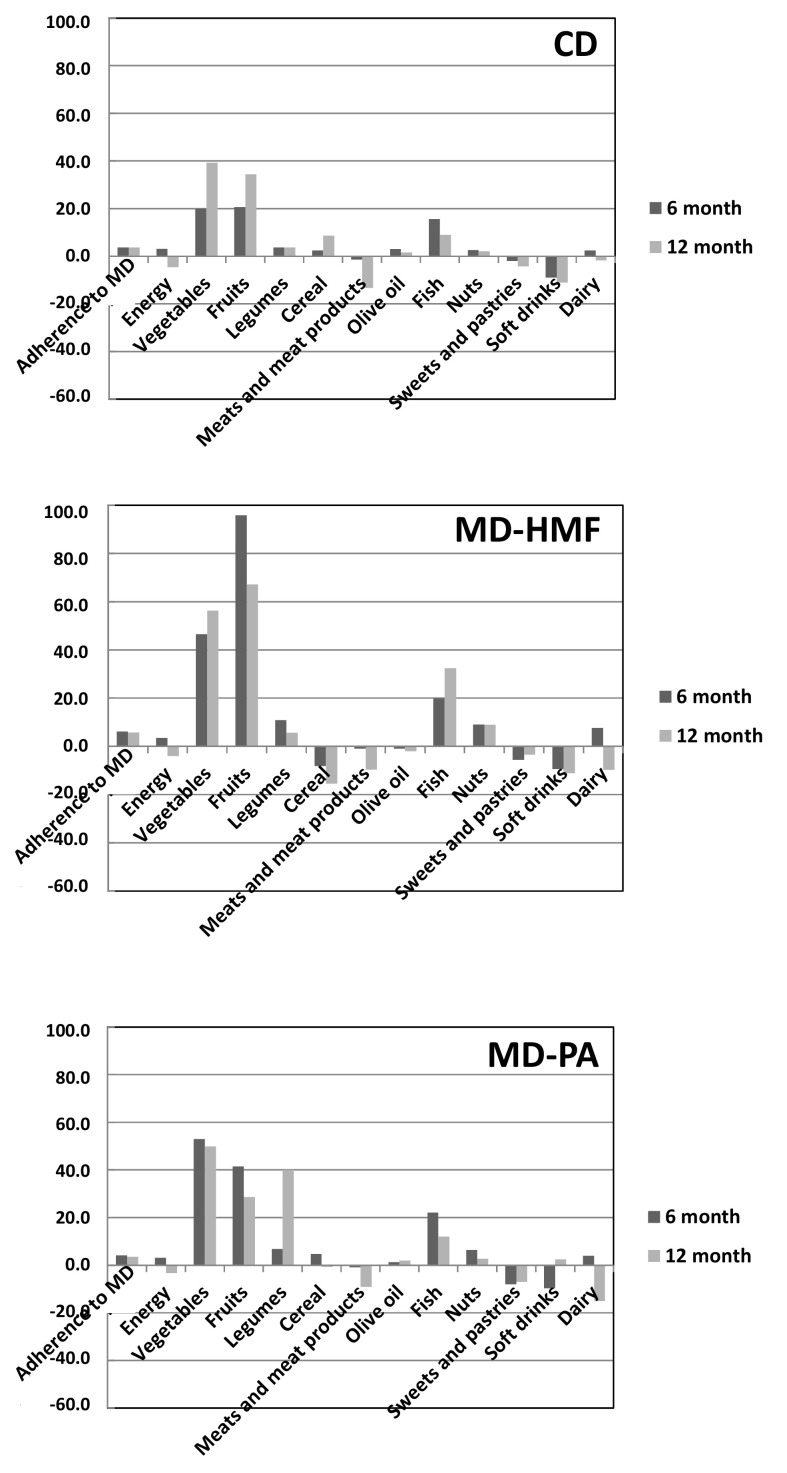
Changes in adherence to MedDiet, energy and nutrients intake at 6 and 12 months follow-up versus baseline. CD: Conventional Diet; MD–HMF: Mediterranean diet–high meal frequency; MD–PA: Mediterranean Diet–physical activity (control group). Energy was represented as kcal × 10^−2^. Meat and dairy intake were represented as g/d per 100 kcal.

Table 3 shows the changes in adherence to the MedDiet at 6 months versus baseline. Energy, legumes, and fish consumption in the 6-month trial improved with no differences between the three groups. Adherence to the MedDiet, fruits and soft drinks improved, with differences between the intervention groups. Differences between the interventions on the adherence to the MD were noticeable, highlighting the increase in adherence between the control group (+3.6 ± 3.0 *p* < 0.001) and the MD–HMF group (+6.2 ± 3.5 *p* < 0.001) with changes in time per group (*p* = 0.002). Regarding fruits, participants improved their consumption, where MD–HMF increased the most (+95.8 ± 116.2 *p* < 0.001), having a significant difference between the control group and MD–HMF (*p* = 0.049). As for soft drinks, MD–HMF reduced their consumption (−9.4 ± 23.3 *p* = 0.011) and by comparing the interventions groups, there was a significant change between the control group with both Mediterranean counterparts (*p* = 0.007). Some differences were found within groups through time; however, no time * group interaction was found. Changes in adherence to the MedDiet, energy and nutrients intake at 6 and 12 months follow-up versus baseline can be visualized clearer in Figure 3.

Table 4 and Figure 2 show changes in anthropometric, metabolic, and liver parameters at 12 months versus baseline, and Table 5 and Figure 3 show changes in adherence to MedDiet, and energy and nutrient intake at 12 months versus baseline, respectively. Obtained results were similar to those obtained after 6 months follow-up.

## 4. Discussion 

In the current randomized trial of participants with metabolic syndrome and NAFLD, after a 6- and 12-month personalized weight loss intervention, subjects significantly reduced intrahepatic fat contents by MRI, and liver stiffness values, with no differences in the three interventions. A possible explanation could be because of the changes that participants went through towards a change in lifestyle, which agrees with a study that reported that beyond the dietary intervention, the lifestyle therapeutic approach to NAFLD may benefit from an improvement in life quality [29]. In the current study, kilocalories were significantly reduced and that could explain why the three interventions worked on the liver parameters. In concordance with the present study, a previous study conducted in Helsinki reported that in general, energy intake is the most relevant factor when it comes to reducing the accumulation of fat in the liver [30]. As previously described, the most effective treatment for NAFLD is weight loss achieved by caloric restriction [31]. 

The three intervention groups worked with no differences between them in 6 months from baseline of the following parameters: For anthropometric parameters, BMI and body weight improved. As for metabolic parameters, ALT, insulin, Hb1Ac, diastolic blood pressure and HDL-cholesterol improved. For dietary parameters, total energy, legumes, and fish consumption increased. A plausible explanation for the amelioration of the anthropometric parameters, besides the reduced energy intake that our study gave to the participants, is that the diets in the current study contained adequate amounts of fiber, which could have contributed to the successful outcome of the interventions. Fiber supplementation increases satiety and decreases eating frequency by stimulating anorexigenic hormones and suppressing the orexigenic hormone ghrelin; this positive effect, combined with its low energy density, has been related to weight loss [32,33]. The improvement of metabolic parameters could be explained by diet amelioration. In a study where the effect of two hypocaloric diets (500 kcal/d less) was observed, it concluded that a reduced total energy intake manages to reduce transaminase levels and insulin resistance in obese patients with fatty liver disease, regardless of the composition of the diet (low carbohydrate, low fat or balanced) [34,35]. Furthermore, the significant increase in fish consumption that participants had was important. The literature shows that fish, particularly fish oil, has a preventive impact against NAFLD [36,37]. Fish and fatty fish, due to their exclusively high content of omega-3 fatty acids, exert their beneficial effect on NAFLD by improving lipidemic and glycemic control, as well as by attenuating inflammation [38]. There is a study that aimed to evaluate the dietary intake of fish and v-3 fatty acids in children, where they proved by a linear regression model the relations between serum ALT and long-chain ω-3 fatty acid intake that tended toward significance by adding factors including demographic, anthropometric, and dietary variables [39]. Additionally, the Toronto study noted a strong inverse relation between EPA and DHA intake and ALT [40]. As for legumes, there is a study that was conducted in Iran that found that a higher intake of total legumes (beans, lentils, and peas) was associated with a lower risk of NAFLD [41]. It is known that dietary intake of legumes is associated with intake of bioactive compounds and vegetable proteins, and lower waist circumference, serum cholesterol, and blood pressure [42]. Legumes have high fiber content and low glycemic index that may help to enhance lipid profiles and reduce lipid peroxidation. Moreover, legumes lowered blood lipids through increasing fat and steroid excretion through feces, as well as short-chain fatty acid synthesis in the colon [43,44,45,46].

In the current study, it was significant that the MedDiet improved participants’ WC. Interestingly, the literature shows that the reduction in participants’ waist size is an important variable. In a cross-sectional study conducted on 1500 individuals living in Tehran, it was assumed that food patterns affect fatty liver by changing waist circumference size, which is an indication of abdominal fat accumulation. The relationship’s power indicates that keeping waist circumference in a normal range may prevent fatty liver, even if a proper diet is not followed [47]. Furthermore, a meta-analysis with 20 structured studies concluded that WC is the strongest anthropometric variable in predicting fatty liver (OR = 3.14; 95% Cl: 2.07–4.77), an even stronger variable than BMI [48]. In the current findings, the MD–HMF group reduced the most. A plausible explanation is that eating more than seven meals per day reduced the risk of obesity compared to eating less than three meals per day; additionally, frequent eaters had lower waist circumference after adjusting for diet and lifestyle [49]. Nevertheless, these data contrast with another study that found no correlation between increased meal frequency and type 2 diabetes risk in women after six years follow up [50]. Another explanation for the improvement in the participants’ WC could be the changes in dietary habits that the MD–HMF group did. There was an increase in consumption of fruits, and a decrease in the consumption of soft drinks compared to the control group. The literature supports these results. A systematic review showed in their findings that increased intake of fruits was inversely associated with WC decrease [51]. Moreover, several studies showed that sweet drinks are linked to an increased risk of obesity, metabolic syndrome, fatty liver, and heart disease, owing to higher calorie intake, as well as rapid and high increases in absorbable sugars [47]. 

The current results showed that adherence to the MedDiet occurred in the three interventions, even though the CD group was not advised to adhere to the MedDiet like the other groups. Interestingly, this group increased the consumption of extra virgin olive oil, even though they were not encouraged to consume olive oil. It seems that subjects themselves preferred olive oil when starting the trial, maybe due to the place the study took place—half of the participants were from the Mediterranean. Moreover, the subjects in the CD, by modifying habits towards a healthier lifestyle, increased adherence. A previous study found that the MedDiet had an effect that was unaffected by other lifestyle changes, and also greater adherence to MedDiet was substantially connected with a reduced degree of insulin resistance, ALT, and NAFLD severity [8]. 

## 5. Strengths and Limitations

The main strength of this study is that liver images were obtained by MRI, which is considered the most sensitive and accurate non-invasive method for quantifying liver fat contents [52]. Participants included in the study were highly compliant with the interventions. On the other hand, the major limitation was the sample size. A bigger sample size could give a more confident answer to which intervention was the best. Another limitation was the estimated values of the FFQ: even after being validated, it might overestimate intake of certain food groups. Thirdly, the study was unable to demonstrate that customized hypocaloric dietary and increased physical activity interventions are more effective at preventing NAFLD than a traditional diet. This could be due to the fact that the Conventional Diet of the AASLD was difficult to carry out in the Mediterranean region; when participants thought of a healthier life, they increased their use of virgin or extra virgin olive oil, nuts, and fish. It was a challenging to make them use other products not from the MedDiet.

## 6. Conclusions

Subjects with NAFLD and MetS after 6 and 12 months of a customized dietary intervention had reduced intrahepatic fat contents, and liver stiffness, despite the intervention the participants went through. All participants ameliorated BMI, insulin, Hb1Ac, diastolic blood pressure, HDL-cholesterol, and ALT, and improved their consumption of total energy, fish, and legumes. Participants in the MD–HMF improved WC, and the consumption of fruits and soft drinks compared to the control group. Customized hypocaloric dietary and enhanced physical activity interventions may be useful to ameliorate NAFLD. Further human studies are needed to enlighten the effects of nutrients in the development of hepatic steatosis and other related diseases.

## Figures and Tables

**Table 1:** Characteristics of participants at baseline.

	CD (*n* = 43)	MD–HMF (*n* = 43)	MD–PA (*n* = 42)	*p* *
Females (*n*; %)	16 (37.2)	14 (32.6)	17 (40.5)	0.875
Females at menopause (*n*; %)	10 (23.3)	6 (14.0)	7 (16.7)	0.672
Age (years) (mean ± SD)	54.1 ± 8.9	52.3 ± 7.1	52.2 ± 5.8	0.648
Currently smoking (*n*; %)	5 (11.6)	7 (16.3)	4 (9.5)	0.361
Hypertension (*n*; %)	14 (32.6)	14 (32.6)	8 (19.0)	0.120
T2DM (*n*; %)	8 (18.6)	10 (23.3)	13 (31.0)	0.268
Hyperlipidemia (*n*; %)	17 (39.5)	18 (41.9)	13 (31.0)	0.683
No physical activity (*n*; %)	20 (46.5)	20 (46.5)	17 (40.5)	0.988

Abbreviations: CD: Conventional Diet; MD–HMF: Mediterranean diet–high meal frequency; MD–PA: Mediterranean Diet–physical activity (control group); SD standard deviation; T2DM: type 2 diabetes mellitus. * Differences between groups: by ANOVA (quantitative variables) and by Chi-square (percentages).

**Table 2:** Changes in 6 months of anthropometric, metabolic, and liver parameters after 6 months of intervention.

		CD (*n* = 43)	MD–HMF (*n* = 43)	MD–PA (*n* = 42)	*p* ^‡^
BMI (kg/m^2^)	Basal	33.6 ± 3.7	33.9 ± 3.9	33.4 ± 3.1	0.145
	6 months	31.4 ± 3.9	31.4 ± 3.8	31.8 ± 3.4	
	Δ	−2.1 ± 1.9 *	−2.5 ± 2.2 *	−1.6 ± 1.7 *	
Body weight (kg)	Basal	92.8 ± 14.4	96.2 ± 13.8	96.8 ± 12.3	0.128
	6 months	86.7 ± 13.6	89.1 ± 13.5	92.2 ± 13.1	
	Δ	−6.0 ± 5.4 *	−7.1 ± 6.3 *	−4.6 ± 4.9 *	
Body fat (%)	Basal	35.7 ± 6.3	35.3 ± 7.2	35.4 ± 7.3	0.308
	6 months	34.4 ± 9.1	34.9 ± 15.9	33.6 ± 8.0	
	Δ	−1.3 ± 7.0	−0.4 ± 14.8	−1.8 ± 2.8 *	
Mass muscle (kg)	Basal	56.7 ± 12.2	58.4 ± 8.4	59.7 ± 10.2	0.136
	6 months	54.8 ± 11.4	56.0 ± 8.5	58.3 ± 10.1	
	Δ	−1.8 ± 2.4 *	−2.4 ± 2.3 *	−1.4 ± 2.1 *	
Waist circumference (cm)	Basal	110.7 ± 9.4	112.1 ± 9.1	112.7 ± 8.5	0.046
	6 months	106.7 ± 18.3	104.8 ± 10.7	108.6 ± 10.0	
	Δ	−4.0 ± 16.3	−7.3 ± 5.8 *^b^	−4.0 ± 6.3 *^b^	
Physical activity (MET)	Basal	2866.8 ± 2292.1	3483.0 ± 2741.1	3529.2 ± 4108.6	0.616
	6 months	3647.6 ± 2577.6	3646.6 ± 2348.8	3833.5 ± 3675.1	
	Δ	780.8 ± 2626.6	163.6 ± 3082.7	304.3 ± 2767.6	
Glucose (mg/dL)	Basal	109.1 ± 22.6	134.5 ± 14.2	121.6 ± 59.7	0.793
	6 months	104.6 ± 34.7	129.0 ± 13.4	109.4 ± 44.4	
	Δ	−4.4 ± 23.6	−5.5 ± 13.8 *	−12.2 ± 44.8	
Hb1Ac (%)	Basal	6.1 ± 1.0	5.9 ± 1.0	6.2 ± 1.6	0.820
	6 months	5.9 ± 0.7	5.7 ± 0.8	5.8 ± 1.2	
	Δ	−0.2 ± 0.7 *	−0.2 ± 0.5 *	−0.4 ± 1.2 *	
HR (beats/min)	Basal	71.7 ± 10.1	69.4 ± 12.2	73.5 ± 12.5	0.786
	6 months	66.3 ± 11.2	66.4 ± 8.9	67.9 ± 10.6	
	Δ	−5.4 ± 9.5 *	−3.0 ± 9.3	−5.6 ± 9.5 *	
SBP (mmHg)	Basal	138.6 ± 15.8	134.5 ± 14.2	134.4 ± 16.1	0.880
	6 months	131.2 ± 17.6	129.0 ± 13.4	131.1 ± 15.8	
	Δ	−7.4 ± 15.3 *	−5.5 ± 13.8 *	−3.3 ± 17.0	
DBP (mmHg)	Basal	85.5 ± 10.4	85.7 ± 8.0	85.3 ± 9.0	0.980
	6 months	81.3 ± 9.1	81.7 ± 7.5	81.8 ± 9.8	
	Δ	−4.1 ± 8.6 *	−4.0 ± 8.9 *	−3.5 ± 8.9 *	
Triglycerides (mg/dL)	Basal	188.2 ± 96.6	201.4 ± 313.1	200.2 ± 122.6	0.096
	6 months	159.2 ± 110.7	128.2 ± 72.1	165.2 ± 108.0	
	Δ	−29.1 ± 103.3	−73.2 ± 290.0	−35.0 ± 128.0	
Total-cholesterol (mg/dL)	Basal	203.0 ± 43.2	199.6 ± 56.0	191.8 ± 39.2	0.661
	6 months	193.8 ± 45.8	184.4 ± 38.8	191.7 ± 42.7	
	Δ	−9.2 ± 28.9 *	−15.1 ± 52.7	−0.1 ± 44.6	
HDL-chol (mg/dL)	Basal	45.5 ± 14.5	45.6 ± 9.2	41.8 ± 9.1	0.664
	6 months	47.8 ± 14.6	48.9 ± 10.8	43.8 ± 9.5	
	Δ	2.3 ± 6.3 *	3.3 ± 8.4 *	2.0 ± 5.0 *	
Intrahepatic fat contents (%)	Basal	16.2 ± 9.3	16.9 ± 13.4	16.5 ± 11.4	0.100
	6 months	10.4 ± 7.5	8.3 ± 8.0	10.7 ± 6.4	
	Δ	−5.8 ± 7.8 *	−8.7 ± 12.4 *	−5.9 ± 10.6 *	
Liver stiffness K-pa	Basal	8.4 ± 1.8	8.6 ± 1.7	8.2 ± 2.6	0.589
	6 months	4.8 ± 2.0	5.2 ± 1.9	5.8 ± 1.8	
	Δ	−3.6 ± 2.5 *	−3.4 ± 1.8 *	−2.4 ± 3.1 *	
AST (mg/dL)	Basal	27.4 ±10.1	26.4 ± 16.6	23.3 ± 10.0	0.609
	6 months	22.9 ± 6.8	23.2 ± 6.7	23.0 ± 15.3	
	Δ	−4.6 ± 8.6 *	−3.2 ± 15.3	−0.3 ± 8.1	
ALT (mg/dL)	Basal	37.5 ± 20.3	38.2 ± 38.7	33.6 ± 27.1	0.464
	6 months	26.5 ± 10.4	25.7 ± 12.8	26.9 ± 24.6	
	Δ	−11.0 ± 16.3 *	−12.5 ± 32.8 *	−6.7 ± 17.3 *	
GGT (mg/dL)	Basal	47.4 ± 30.3	58.3 ± 81.0	37.3 ± 17.4	0.591
	6 months	33.7 ± 22.9	47.7 ± 77.8	31.7 ± 19.6	
	Δ	−13.7 ± 22.1 *	−10.6 ± 67.2	−5.6 ± 11.3 *	

Abbreviations: ALT: alanine aminotransferase; AST: aspartate aminotransferase; BMI: body mass index; CD: Conventional Diet; ∆: change between basal and 6-month data; DBP: Diastolic blood pressure; GGT: gamma-glutamyl transferase; HbA1c: glycated hemoglobin; HDL-c: High density lipoprotein-cholesterol; HR: heart rate; kPa: kilopascals; MD–HMF: Mediterranean diet–high meal frequency; MD–PA: Mediterranean Diet–physical activity (control group); MedDiet: Mediterranean diet; METs: metabolic equivalents; SBP: systolic blood pressure; TG: triglycerides; Total-c: total cholesterol. Data are expressed as mean ± standard deviation (SD). ∆: delta * *p* < 0.05. ^‡^ Differences between the three intervention groups at 6 months after adjustment for baseline values and age by ANCOVA. Post hoc test by Bonferroni: “^b^: differences between MD–PA and MD–HMF”.

**Table 3:** Changes in adherence to the Mediterranean diet after 6 months of intervention.

		CD (*n* = 43)	MD–HMF (*n* = 43)	MD–PA (*n* = 42)	*p* ^‡^
Adherence to MedDiet	Basal	7.3 ± 2.6	6.9 ± 2.7	7.7 ± 2.3	0.002
	6 months	10.9 ± 2.8	13.1 ± 2.7	11.8 ± 2.8	
	Δ	3.6 ± 3.0 *^a^	6.2 ± 3.5 *^a^	4.1 ± 3.0 *	
Energy (kcal/d)	Basal	2481.2 ± 633.0	2311.7 ± 980.1	2437.0 ± 697.2	0.529
	6 months	2166.3 ± 676.9	1964.8 ± 519.2	2126.1 ± 626.6	
	Δ	−314.9 ± 762.8 *	−346.9 ± 947.2 *	−310.9 ± 747.8 *	
Vegetables (g/d) per 1000 kcal	Basal	144.2 ± 111.5	138.4 ± 74.5	131.2 ± 82.7	0.229
	6 months	164.1 ± 72.9	184.8 ± 88.6	184.2 ± 85.7	
	Δ	19.9 ± 123.4	46.5 ± 88.6 *	53.0 ± 66.5 *	
Fruits (g/d) per 1000 kcal	Basal	137.7 ± 75.7	110.2 ± 80.9	131.7 ± 96.8	0.049
	6 months	158.3 ± 93.6	206.0 ± 121.7	173.2 ± 109.5	
	Δ	20.6 ± 93.8 ^a^	95.8 ± 116.2 *^a^	41.5 ± 95.7 *	
Legumes (g/d) per 1000 kcal	Basal	9.5 ± 5.7	10.2 ± 6.5	9.1 ± 5.2	0.113
	6 months	13.2 ± 9.2	21.1 ± 16.2	15.9 ± 10.4	
	Δ	3.7 ± 9.5 *	10.9 ± 16.1 *	6.8 ± 10.0 *	
Cereal (g/d) per 1000 kcal	Basal	64.1 ± 27.7	62.0 ± 33.7	57.7 ± 31.6	0.070
	6 months	66.5 ± 31.8	53.9 ± 53.9	62.4 ± 24.0	
	Δ	2.4 ± 39.9	−8.1 ± 43.1	4.7 ± 37.8	
Meats and meat products (g/d) per 1000 kcal	Basal	81.3 ± 37.6	79.2 ± 29.0	78.3 ± 34.3	0.256
	6 months	67.0 ± 24.3	70.1 ± 32.9	69.2 ± 29.7	
	Δ	−14.3 ± 41.8 *	−9.1 ± 28.4 *	−9.1 ± 29.9	
Olive oil (g/d) per 1000 kcal	Basal	12.9 ± 6.2	14.7 ± 9.5	12.9 ± 6.2	0.406
	6 months	15.9 ± 8.7	13.8 ± 7.9	14.1 ± 8.4	
	Δ	3.0 ± 8.5 *	−0.9 ± 10.4	1.3 ± 9.2	
Fish (g/d) per 1000 kcal	Basal	41.1 ± 26.4	36.6 ± 24.0	48.5 ± 29.3	0.011
	6 months	56.7 ± 42.5	56.5 ± 27.2	70.6 ± 38.3	
	Δ	15.6 ± 41.1 *	19.9 ± 32.4 *	22.1 ± 32.9 *	
Nuts (g/d) per 1000 kcal	Basal	5.3 ± 6.3	4.6 ± 5.5	6.1 ± 7.4	0.452
	6 months	7.8 ± 9.9	13.7 ± 13.9	12.5 ± 10.2	
	Δ	2.6 ± 9.9	9.1 ± 14.0 *	6.4 ± 10.3 *	
Sweets and pastries (g/d) per 1000 kcal	Basal	10.1 ± 11.2	10.9 ± 15.4	13.7 ± 14.9	0.684
	6 months	8.1 ± 11.6	5.3 ± 7.9	5.8 ± 8.9	
	Δ	−2.0 ± 12.2	−5.6 ± 18.0 *	−8.0 ± 15.5 *	
Soft Drinks (mL/d) per 1000 kcal	Basal	14.6 ± 33.5	10.7 ± 23.2	10.3 ± 36.6	0.007
	6 months	5.6 ± 13.1	1.3 ± 2.8	0.5 ± 1.8	
	Δ	−9.0 ± 85.1 ^a,c^	−9.4 ± 23.3 *^a^	−9.8 ± 36.5 ^c^	
Dairy (mL/d) per 1000 kcal	Basal	156.7 ± 103.1	126.8 ± 63.8	136.2 ± 88.1	0.286
	6 months	181.4 ± 115.2	203.2 ±128.5	176.0 ± 112.2	
	Δ	24.7 ± 92.0	76.4 ± 122.9 *	39.8 ± 107.0 *	

Abbreviations: Adherence to MedDiet: Adherence to Mediterranean Diet; ∆: delta; CD: Conventional Diet; MD–HMF: Mediterranean diet–high meal frequency; MD–PA: Mediterranean Diet–physical activity (control group). Data are expressed as mean ± standard deviation. ∆: delta * *p* < 0.05; ^‡^ Differences between the three intervention groups at 6 months after adjustment for baseline values and age by ANCOVA. Post hoc test by Bonferroni: “^a^: differences between CD and MD–HMF groups”; “^c^: differences between CD and MD–PA”.

**Table 4:** Changes in anthropometric, metabolic, and liver parameters after 12 months of intervention.

		CD (*n* = 43)	MD–HMF (*n* = 43)	MD–PA (*n* = 42)	*p* ^‡^
BMI (kg/m^2^)	Basal	33.6 ± 3.7	34.3 ± 4.0	33.4 ± 3.1	0.006
	12 months	31.9 ± 4.1	31.7 ± 4.3	31.8 ± 3.5	
	Δ	−1.7 ± 1.8 *^a^	−2.6 ± 2.2 *^a.b^	−1.6 ± 1.8 *^b^	
Body weight (kg)	Basal	92.7 ± 14.4	96.3 ± 13.8	95.3 ± 12.3	0.031
	12 months	88.4 ± 14.4	89.5 ± 14.4	91.6 ± 13.0	
	Δ	−4.3 ± 5.5 *	−6.8 ± 6.4 *^b^	−3.7 ± 5.0 *^b^	
Body fat (%)	Basal	35.2 ± 6.3	35.4 ± 7.2	35.7 ± 7.3	0.737
	12 months	33.3 ± 6.1	34.2 ± 9.8	34.9 ± 8.2	
	Δ	−1.9 ± 2.6 *	−1.2 ± 8.4	−1.2 ± 7.2	
Mass muscle (kg)	Basal	57.6 ± 12.2	59.5 ± 8.4	58.7 ± 10.2	0.099
	12 months	56.1 ± 11.1	56.1 ± 10.4	57.6 ± 11.0	
	Δ	−1.6 ± 2.5 *	−3.3 ± 6.6 *	−1.1 ± 2.0 *	
Waist circumference (cm)	Basal	110.7 ± 9.4	112.1 ± 9.1	112.7 ± 8.5	0.044
	12 months	105.0 ± 10.25	104.8 ± 12.0	107.3 ± 9.9	
	Δ	−5.2 ± 6.3 *	−7.3 ± 6.2 *	−4.1 ± 6.0 *	
Physical activity (MET)	Basal	2750.2 ± 2327.7	3772.4 ± 3275.0	3615.0 ± 4171.1	0.063
	12 months	4357.8 ± 4305.6	3440.4 ± 3101.9	4333.1 ± 3849.8	
	Δ	1607.6 ± 3819.3 *	−331.9 ± 3278.9	718.1 ± 4189.2	
Glucose (mg/dL)	Basal	114.3 ± 36.0	108.7 ± 26.4	118.2 ± 51.9	0.546
	12 months	108.2 ± 29.8	100.7 ± 23.1	105.1 ± 18.2	
	Δ	−6.0 ± 16.3 *	−8.0 ± 20.2 *	−13.1 ± 46.0	
Hb1Ac (%)	Basal	6.1 ± 1.0	5.9 ± 1.0	6.1 ± 1.5	0.276
	12 months	6.0 ± 1.0	5.7 ± 0.7	5.7 ± 0.6	
	Δ	−0.1 ± 0.4	−0.2 ± 0.6	−0.4 ± 1.3	
HR (beats/min)	Basal	69.4 ± 11.4	67.0 ± 11.4	67.3 ± 8.9	0.541
	12 months	63.8 ± 11.1	60.6 ± 11.1	63.7 ± 11.0	
	Δ	−5.6 ± 7.8 *	−6.4 ± 7.4 *	−3.6 ± 8.3	
SBP (mmHg)	Basal	136.3 ± 12.7	133.3 ± 14.2	131.9 ± 13.6	0.472
	12 months	127.0 ± 15.0	129.1 ± 20.6	125.5 ± 16.6	
	Δ	−9.3 ± 12.9 *	−4.2 ± 15.3	−6.4 ± 14.0	
DBP (mmHg)	Basal	87.7 ± 10.4	88.1 ± 6.7	87.9 ± 6.6	0.875
	12 months	80.5 ± 9.1	82.2 ± 8.4	80.9 ± 8.2	
	Δ	−7.2 ± 7.5 *	−5.9 ± 10.3 *	−7.0 ± 8.5 *	
Triglycerides (mg/dL)	Basal	186.5 ± 97.8	207.6 ± 330.1	184.4 ± 115.2	0.478
	12 months	161.7 ± 108.7	132.0 ± 64.7	148.1 ± 76.5	
	Δ	−24.8 ± 85.5	−75.6 ± 317.0	−36.3 ± 119.2	
Total- cholesterol (mg/dL)	Basal	202.7 ± 43.2	202.7 ± 57.1	196.0 ± 40.6	0.305
	12 months	195.0 ± 46.8	183.1 ± 39.6	191.6 ± 40.7	
	Δ	−7.7 ± 33.8	−19.6 ± 63.6 *	−4.4 ± 36.8	
HDL-chol (mg/dL)	Basal	45.5 ± 14.6	46.1 ± 9.0	43.0 ± 9.3	0.560
	12 months	47.3 ± 14.2	49.5 ± 11.4	45.4 ± 11.9	
	Δ	1.8 ± 7.1	3.3 ± 7.4 *	2.4 ± 6.2 *	
Intraliver fat contents (%)	Basal	14.5 ± 10.1	12.0 ± 12.1	13.5 ± 11.8	0.372
	12 months	12.6 ± 9.1	7.1 ± 5.8	10.6 ± 7.7	
	Δ	−1.8 ± 7.6	−4.9 ± 10.6 *	−2.9 ± 11.3	
Liver stiffness K-pa	Basal	5.3 ± 1.7	5.3 ± 1.9	5.3 ± 2.2	0.300
	12 months	5.0 ± 1.7	4.8 ± 1.5	5.7 ± 2.4	
	Δ	−0.3 ± 2.1	−0.4 ± 2.2	−0.4 ± 2.8	
AST (mg/dL)	Basal	28.4 ± 10.1	27.7 ± 17.5	23.9 ± 9.0	0.041
	12 months	27.4 ± 17.1	20.9 ± 5.6	22.8 ± 8.8	
	Δ	−1.0 ± 16.0 ^c^	−6.8 ± 16.2	−1.1 ± 7.2 *^c^	
ALT (mg/dL)	Basal	37.5 ± 20.4	39.4 ± 40.6	33.6 ± 23.1	0.240
	12 months	33.4 ± 25.7	25.0 ± 13.0	25.7 ± 16.3	
	Δ	−4.2 ± 28.3	−14.4 ± 36.4 *	−8.0 ± 15.1 *	
GGT (mg/dL)	Basal	47.1 ± 30.3	51.7 ± 71.2	37.5 ± 18.0	0.348
	12 months	43.8 ± 44.3	36.3 ± 37.5	31.2 ± 16.4	
	Δ	−3.3 ± 36.3	−15.4 ± 56.4	−6.3 ± 13.0 *	

Abbreviations: ALT: alanine aminotransferase; AST: aspartate aminotransferase; BMI: body mass index; CD: Conventional Diet; ∆: change between basal and 12-month data; DBP: Diastolic blood pressure; GGT: gamma-glutamyltransferase; HbA1c: glycated hemoglobin; HDL-c: High density lipoprotein-cholesterol; HR: heart rate; kPa: kilopascals; MD–HMF: Mediterranean diet–high meal frequency; MD–PA: Mediterranean Diet–physical activity (control group); MedDiet: Mediterranean diet; METs: metabolic equivalents; SBP: systolic blood pressure; TG: triglycerides; Total-c: total cholesterol. Data are expressed as mean ± standard deviation (SD). ∆: delta * *p* < 0.05 ^‡^ Differences between the three intervention groups at 12 months after adjustment for baseline values and age by ANCOVA. Post hoc test by Bonferroni: “^a^: differences between CD and MD–HMF groups”; “^b^: differences between MD–PA and MD–HMF”; “^c^: differences between CD and MD–PA”.

**Table 5:** Changes in adherence to the Mediterranean diet after 12 months of intervention.

		CD (*n* = 43)	MD–HMF (*n* = 43)	MD–PA (*n* = 42)	*p* ^‡^
Adherence to MedDiet	Basal	7.2 ± 2.6	7.0 ± 2.7	7.9 ± 2.3	0.001
	12 months	10.8 ± 2.6	12.7 ± 2.6	11.5 ± 2.3	
	Δ	3.6 ± 2.9 *^c^	5.7 ± 3.3 *	3.5 ± 2.6 *^c^	
Energy (kcal/day)	Basal	2500.9 ± 643.9	2372.4 ± 992.7	2364.8 ± 695.9	0.845
	12 months	2035.9 ± 628.1	1971.4 ± 893.7	2036.1 ± 514.8	
	Δ	−464.9 ± 723.0 *	−401.0 ± 1305.4 *	−328.7 ± 617.6 *	
Vegetables (g/day) per 1000 kcal	Basal	139.5 ± 110.7	133.0 ± 65.5	143.0 ± 87.7	0.695
	12 months	178.8 ± 120.1	189.3 ± 81.7	192.7 ± 112.6	
	Δ	39.3 ± 100.2 *	56.3 ± 68.9 *	49.8 ± 101.7 *	
Fruits (g/day) per 1000 kcal	Basal	131.2 ± 77.1	114.1 ± 82.3	138.8 ± 97.2	0.047
	12 months	165.5 ± 121.9	181.3 ± 117.0	167.4 ± 106.7	
	Δ	34.4 ± 121.3	67.2 ± 133.1 *	28.6 ± 86.5	
Legumes (g/day) per 1000 kcal	Basal	9.2 ± 5.6	10.0 ± 6.4	9.7 ± 5.6	0.604
	12 months	12.9 ± 9.4	15.6 ± 10.9	13.7 ± 8.5	
	Δ	3.7 ± 8.7 *	5.6 ± 9.6 *	4.0 ± 9.5 *	
Cereal (g/day) per 1000 kcal	Basal	65.4 ± 29.9	66.1 ± 33.6	54.5 ± 28.5	0.031
	12 months	74.1 ± 39.1	50.6 ± 29.7	53.8 ± 22.5	
	Δ	8.7 ± 46.5 ^c^	−15.4 ± 44.1 *	−0.7 ± 25.2 ^c^	
Meats and meat products (g/day) per 1000 kcal	Basal	198.7 ± 90.4	173.9 ± 63.5	174.0 ± 73.4	0.063
	12 months	65.4 ± 39.9	77.8 ± 50.5	83.4 ± 35.5	
	Δ	−133.3 ± 102.0 *	−96.1 ± 77.7 *	−90.7 ± 64.2 *	
Olive oil (g/d) per 1000 kcal	Basal	13.5 ± 7.0	14.9 ± 9.4	13.1 ± 5.8	0.149
	12 months	15.1 ± 8.5	12.9 ± 7.5	15.0 ± 6.9	
	Δ	1.6 ± 10.0	−2.0 ± 10.2	1.9 ± 8.2	
Fish (g/day) per 1000 kcal	Basal	40.5 ± 26.0	34.9 ± 21.5	51.9 ± 31.3	0.006
	12 months	49.5 ± 31.6	67.3 ± 38.7	63.9 ± 30.8	
	Δ	9.0 ± 30.9 ^c^	32.4 ± 39.8 *	12.0 ± 31.1 *^c^	
Nuts (g/day) per 1000 kcal	Basal	5.1 ± 6.3	4.3 ± 5.5	6.3 ± 7.7	0.001
	12 months	7.3 ± 7.6	13.4 ± 11.5	9.0 ± 8.1	
	Δ	2.1 ± 6.4 *^c^	9.0 ± 12.0 *	2.7 ± 6.7 *^c^	
Sweets and pastries (g/day) per 1000 kcal	Basal	12.8 ± 16.5	11.5 ± 15.0	12.2 ± 15.2	0.666
	12 months	8.4 ± 14.3	8.1 ± 18.8	5.2 ± 7.0	
	Δ	−4.3 ± 15.3	−3.4 ± 18.9	−7.0 ± 14.9 *	
Soft Drinks (mL/day) per 1000 kcal	Basal	15.9 ± 34.1	12.2 ± 24.6	11.0 ± 41.0	0.071
	12 months	4.7 ± 12.6	1.1 ± 2.5	13.6 ± 53.8	
	Δ	−11.1 ± 30.9 *	−11.1 ± 24.8 *	2.5 ± 60.2	
Dairy (mL/day) per 1000 kcal	Basal	361.3 ± 190.0	312.2 ± 164.1	297.3 ± 241.7	0.163
	12 months	180.0 ± 175.1	214.9 ± 118.5	146.5 ± 98.6	
	Δ	−181.3 ± 229.9 *	−97.3 ± 140.1 *	−150.8 ± 225.5 *	

Abbreviations: Adherence to MedDiet: Adherence to Mediterranean Diet; ∆: change between basal and 12-month data; CD: Conventional Diet; MD–HMF: Mediterranean diet–high meal frequency; MD–PA: Mediterranean Diet–physical activity (control group). Data are expressed as mean ± standard deviation. ∆: delta * *p* < 0.05; ^‡^ Differences between the three intervention groups at 12 months after adjustment for baseline values and age by ANCOVA, Post hoc test by Bonferroni. “^c^: differences between CD and MD–PA”.

## Data Availability

There are restrictions on the availability of data for this trial, due to the signed consent agreements around data sharing, which only allow access to external researchers for studies following the project’s purposes. Requestors wishing to access the trial data used in this study can make a request to pep.tur@uib.es.

## References

[1] Wong R.J., Aguilar M., Cheung R., Perumpail R.B., Harrison S.A., Younossi Z.M. (2015). Non-alcoholic steatohepatitis is the second leading etiology of liver disease among adults awaiting liver transplantation in the United States. Gastroenterology.

[2] Younossi Z.M., Marchesini G., Pinto-Cortez H., Petta S. (2019). Epidemiology of non-alcoholic fatty liver disease and non-alcoholic steatohepatitis: Implications for liver transplantation. Transplantation.

[3] Buzzetti E., Pinzani M., Tsochatzis E.A. (2016). The multiple-hit pathogenesis of non-alcoholic fatty liver disease (NAFLD). Metabolism.

[4] Gadiparthi C., Spatz M., Greenberg S., Iqbal U., Kanna S., Satapathy S.K., Broder A., Ahmed A. (2020). NAFLD epidemiology, emerging pharmacotherapy, liver transplantation implications and the trends in the United States. J. Clin. Trans. Hepatol..

[5] European Association for the Study of the Liver (EASL), European Association for the Study of Diabetes (EASD), European Association for the Study of Obesity (EASO) (2016). EASL-EASD-EASO clinical practice guidelines for the management of non-alcoholic fatty liver disease. J. Hepatol..

[6] Sotos-Prieto M., Ortolá R., Ruiz-Canela M., Garcia-Esquinas E., Martínez-Gómez D., Lopez-Garcia E., Martínez-González M.A., Rodriguez-Artalejo F. (2021). Association between the Mediterranean lifestyle, metabolic syndrome and mortality: A whole-country cohort in Spain. Cardiovasc. Diabetol..

[7] Anania C., Perla F.M., Olivero F., Pacifico L., Chiesa C. (2018). Mediterranean diet and nonalcoholic fatty liver disease. World J. Gastroenterol..

[8] Kontogianni M.D., Tileli N., Margariti A., Georgoulis M., Deutsch M., Tiniakos D., Fragopoulou E., Zafiropoulou R., Manios Y., Papatheodoridis G. (2014). Adherence to the Mediterranean diet is associated with the severity of non-alcoholic fatty liver disease. Clin. Nutr..

[9] International Diabetic Federation (IDF) Consensus Statement—The IDF Consensus Worldwide Definition of the Metabolic Syndrome. 2006. 24p. https://www.idf.org/e-library/consensus-statements/60-idfconsensus-worldwidedefinitionof-the-metabolic-syndrome.html.

[10] Chalasani N., Younossi Z., Lavine J.E., Diehl A.M., Brunt E.M., Cusi K., Charlton M., Sanyal A.J. (2012). The diagnosis and management of non-alcoholic fatty liver disease: Practice guideline by the American Association for the Study of Liver Diseases, American College of Gastroenterology, and the American Gastroenterological Association. Am. J. Gastroenterol..

[11] Houttu V., Csader S., Nieuwdorp M., Holleboom A.G., Schwab U. (2021). Dietary Interventions in Patients with Non-alcoholic Fatty Liver Disease: A Systematic Review and Meta-Analysis. Front. Nutr..

[12] Chalasani N., Younossi Z., Lavine J.E., Diehl A.M., Brunt E.M., Charlton M., Cusi K., Rinella M., Sanyal J.A. (2018). The diagnosis and management of non-alcoholic fatty liver disease: Practice guidance from the American Association for the Study of Liver Diseases. Hepatology.

[13] U.S. Department of Health and Human Services, U.S. Department of Agriculture (2015). 2015–2020 Dietary Guidelines for Americans. http://health.gov/dietaryguidelines/2015/guidelines/.

[14] World Health Organization (WHO) Global recommendations on physical activity for health. Geneva World Heal Organ. 2010. 60p. https://www.who.int/publications/i/item/9789241599979.

[15] De La Iglesia R., Lopez-Legarrea P., Abete I., Bondia-Pons I., Navas-Carretero S., Forga L., Martinez J.A., Zulet M.A. (2014). A new dietary strategy for long-term treatment of the metabolic syndrome is compared with the American heart association (AHA) guidelines: The MEtabolic Syndrome REduction in NAvarra (RESMENA) project. Br. J. Nutr..

[16] Zulet A., Bondia-Pons I., Abete I., de la Iglesia R., López-Legarrea P., Forga L., Navas-Carretero S. (2011). The reduction of the metabolyc syndrome in Navarra-Spain (RESMENA-S) study; A multidisciplinary strategy based on chrononutrition and nutritional education, together with dietetic and psychological control. Nutr. Hosp..

[17] Estruch R., Ros E., Salas-Salvadó J., Covas M.I., Corella D., Arós F., Gómez-Gracia E., Ruiz-Gutiérrez V., Fiol M., Lapetra J. (2013). Primary prevention of cardiovascular disease with a Mediterranean diet. N. Engl. J. Med..

[18] Mascaró C.M., Bouzas C., Tur J.A. (2021). Association between Non-Alcoholic Fatty Liver Disease and Mediterranean Lifestyle: A Systematic Review. Nutrients.

[19] Sargeant J.A., Bawden S., Aithal G.P., Simpson E.J., Macdonald I.A., Turner M.C., Cegielski J., Smith K., Dorling J., Gowland P.A. (2018). Effects of sprint interval training on ectopic lipids and tissue-specific insulin sensitivity in men with non-alcoholic fatty liver disease. Eur. J. Appl. Physiol..

[20] Fernández-Ballart J.D., Piñol J.L., Zazpe I., Corella D., Carrasco P., Toledo E., Perez-Bauer M., Martínez-González M.Á., Salas-Salvadó J., Martín-Moreno J.M. (2010). Relative validity of a semi-quantitative food-frequency questionnaire in an elderly Mediterranean population of Spain. Br. J. Nutr..

[21] Moreiras O., Carbajal A., Cabrera L., Cuadrado C. (2018). Food Composition Tables (Spanish).

[22] Schröder H., Fitó M., Estruch R., Martínez-González M.A., Corella D., Salas-Salvadó J., Lamuela-Raventós R., Ros E., Salaverría I., Fiol M. (2011). A Short screener is valid for assessing Mediterranean diet adherence among older Spanish men and women. J. Nutr..

[23] Elosua R., Garcia M., Aguilar A., Molina L., Covas M.I., Marrugat J. (2000). Validation of the Minnesota leisure time physical activity questionnaire in Spanish women. Med. Sci. Sports Exerc..

[24] Elosua R., Marrugat J., Molina L., Pons S., Pujol E. (1994). Validation of the Minnesota leisure time physical activity questionnaire in Spanish men. Am. J. Epidemiol..

[25] Friedewald W.T., Levy R.I., Fredrickson D.S. (1972). Estimation of the concentration of low-density lipoprotein cholesterol in plasma, without use of the preparative ultracentrifuge. Clin. Chem..

[26] Tang A., Tan J., Sun M., Hamilton G., Bydder M., Wolfson T., Gamst A.C., Middleton M., Brunt E.M., Loomba R. (2013). Non-alcoholic fatty liver disease: MR imaging of liver proton density fat fraction to assess hepatic steatosis. Radiology.

[27] Singh D., Das C.D., Baruah M.P. (2013). Imaging of non-alcoholic fatty liver disease: A road less travelled. Indian J. Endocrinol. Metab..

[28] Gargallo Fernández M., Basulto Marset J., Breton Lesmes I., Quiles Izquierdo J., Formiguera Sala X., Salas-Salvadó J. (2012). Recomendaciones nutricionales basadas en la evidencia para la prevención y el tratamiento del sobrepeso y la obesidad en adultos (consenso FESNAD-SEEDO). metodología y resumen ejecutivo (I/III). Nutr. Hosp..

[29] Pani A., Giossi R., Menichelli D., Fittipaldo V.A., Agnelli F., Inglese E., Romandini A., Roncato R., Pintaudi B., Del Sole F. (2020). Inositol and Non-Alcoholic Fatty Liver Disease: A Systematic Review on Deficiencies and Supplementation. Nutrients.

[30] Yki-Järvinen H. (2015). Nutritional modulation of non-alcoholic fatty liver disease and insulin resistance. Nutrients.

[31] Hannah W.N., Harrison S.A. (2016). Effect of Weight Loss, Diet, Exercise, and Bariatric Surgery on Nonalcoholic Fatty Liver Disease. Clin. Liver Dis..

[32] Solah V., Kerr D.A., Hunt W., Johnson S., Boushey C.J., Delp E.J., Meng X., Gahler J.R., James A.P., Mukhtar A.S. (2017). Effect of fibre supplementation on body weight and composition, frequency of eating and dietary choice in overweight individuals. Nutrients.

[33] Parnell J.A., Reimer R.A. (2009). Weight loss during oligofructose supplementation is associated with decreased ghrelin and increased peptide YY in overweight and obese adults. Am. J. Clin. Nutr..

[34] Aller R., de Luis D.A., Izaola O., de la Fuente B., Bachiller R. (2014). Effect of a high monounsaturated vs high polyunsaturated fat hypocaloric diets in nonalcoholic fatty liver disease. Eur. Rev. Med. Pharmacol. Sci..

[35] De Luis D.A., Aller R., Izaola O., Gonzalez Sagrado M., Conde R. (2010). Effect of two different hypocaloric diets in transaminases and insulin resistance in nonalcoholic fatty liver disease and obese patients. Nutr. Hosp..

[36] Mouzaki M., Allard J.P. (2012). The role of nutrients in the development, progression, and treatment of non-alcoholic fatty liver disease. J. Clin. Gastroenterol..

[37] Parker H.M., Johnson N.A., Burdon C.A., Cohn J.S., O’Connor H.T., George J. (2012). Omega-3 supplementation, and non-alcoholic fatty liver disease: A systematic review and meta-analysis. J. Hepatol..

[38] Kalafati I.P., Dimitriou M., Borsa D., Vlachogiannakos J., Revenas K., Kokkinos A., Ladas S.D., Dedoussis G.V. (2019). Fish intake interacts with TM6SF2 gene variant to affect NAFLD risk: Results of a case-control study. Eur. J. Nutr..

[39] St-Jules D.E., Watters C.A., Brunt E.M., Wilkens L.R., Novotny R., Belt P., Lavine J.E. (2013). Estimation of fish and ω-3 fatty acid intake in pediatric nonalcoholic fatty liver disease. J. Pediatr. Gastroenterol. Nutr..

[40] Mager D.R., Patterson C., So S., Rogenstein C.D., Wykes L.J., A Roberts E. (2010). Dietary and physical activity patterns in children with fatty liver. Eur. J. Clin. Nutr..

[41] Bahrami A., Teymoori F., Eslamparast T., Sohrab G., Hejazi E., Poustchi H., Hekmatdoost A. (2019). Legume intake and risk of nonalcoholic fatty liver disease. Indian J. Gastroenterol..

[42] Papanikolaou Y., Fulgoni V.L. (2008). Bean consumption is associated with greater nutrient intake, reduced systolic blood pressure, lower body weight, and a smaller waist circumference in adults: Results from the National Health and Nutrition Examination Survey 1999–2002. J. Am. Coll. Nutr..

[43] Zhang Z., Lanza E., Kris-Etherton P.M., Colburn N.H., Bagshaw D., Rovine M.J., Ulbrecht J.S., Bobe G., Chapkin R.S., Hartman T.J. (2010). A high legume low glycemic index diet improves serum lipid profiles in men. Lipids.

[44] Birketvedt G.S., Travis A., Langbakk B., Florholmen J.R. (2002). Dietary supplementation with bean extract improves lipid profile in overweight and obese subjects. Nutrition.

[45] Macarulla M.T., Medina C., De Diego M.A., Chávarri M., Zulet M., Martínez J.A., Nöel-Suberville C., Higueret P., Portillo M.P. (2001). Effects of the whole seed and a protein isolate of faba bean (Vicia faba) on the cholesterol metabolism of hypercholesterolaemic rats. Br. J. Nutr..

[46] Finley J.W., Burrell J.B., Reeves P.G. (2007). Pinto bean consumption changes SCFA profiles in fecal fermentations, bacterial populations of the lower bowel, and lipid profiles in blood of humans. J. Nutr..

[47] Ghaemi A., Hosseini N., Osati S., NaghiZadeh M.M., Dehghan A., Ehrampoush E., Honarvar B., Homayounfar R. (2018). Waist circumference is a mediator of dietary pattern in Non-alcoholic fatty liver disease. Sci. Rep..

[48] Pang Q., Zhang J.Y., Song S.D., Qu K., Xu X.S., Liu S.S., Liu C. (2015). Central obesity and nonalcoholic fatty liver disease risk after adjusting for body mass index. World J. Gastroenterol..

[49] Paoli A., Tinsley G., Bianco A., Moro T. (2019). The influence of meal frequency and timing on health in humans: The role of fasting. Nutrients.

[50] Mekary R.A., Giovannucci E., Cahill L., Willett W.C., van Dam R.M., Hu F.B. (2013). Eating patterns and type 2 diabetes risk in older women: Breakfast consumption and eating frequency. Am. J. Clin. Nutr..

[51] Schwingshackl L., Hoffmann G., Kalle-Uhlmann T., Arregui M., Buijsse B., Boeing H. (2015). Fruit and vegetable consumption and changes in anthropometric variables in adult populations: A systematic review and meta-analysis of prospective cohort studies. PLoS ONE.

[52] Lv S., Jiang S., Liu S., Dong Q., Xin Y., Xuan S. (2018). Noninvasive quantitative detection methods of liver fat content in non-alcoholic fatty liver disease. J. Clin. Transl. Hepatol..

